# Left atrial myxoma presenting with acute pulmonary edema and syncope in a middle-aged black African woman: a case report

**DOI:** 10.11604/pamj.2023.46.31.41414

**Published:** 2023-09-21

**Authors:** Kasiemobi Eberechukwu Uchime, Oladipo Ayoola Olanipekun, Olamide Nelson Ogidan, Stephen Olawale Oguntola, Okechukwu Obumneme Ezekpo, Olaniyi Abiodun Oluwafemi Olatunde, Akinola Akinmade

**Affiliations:** 1 Department of Anatomic Pathology and Forensic Medicine, Afe Babalola University Ado-Ekiti Teaching Hospital (ABUADTH), Ekiti State, Nigeria,; 2 Department of Internal Medicine, Afe Babalola University Ado-Ekiti Teaching Hospital (ABUADTH), Ekiti State, Nigeria,; 3 Department of Radiology, Afe Babalola University Ado-Ekiti Teaching Hospital (ABUADTH), Ekiti State, Nigeria,; 4 Department of Surgery, Afe Babalola University Ado-Ekiti Teaching Hospital (ABUADTH), Ekiti State, Nigeria

**Keywords:** Heart, neoplasm, myxoma, case report

## Abstract

Cardiac myxoma is a very rare benign cardiac neoplasm. Its annual incidence globally is between 0.5 to 1 case per one million individuals. It has a 0.03% prevalence rate in the general population. It commonly occurs in the left atrium, but can also be located in the other heart chambers. Its clinical presentations are variable, non-specific, and can mimic various cardiovascular and systemic diseases, posing a diagnostic dilemma. Thus, a high index of suspicion with appropriate use of radiologic and laboratory diagnostic tools is essential for its accurate diagnosis and management. The diagnosis and management of a rare case of left atrial myxoma in a middle-aged African woman who presented with heart failure-like symptoms, features of acute pulmonary edema, and syncope is presented in this literature. The diagnosis was suspected following echocardiography. The tumor was surgically excised, and the diagnosis was confirmed histopathologically. The patient´s post-operative condition has been excellent.

## Introduction

Cardiac myxoma, though constituting 86.8% of all benign cardiac tumors, is a very rare neoplasm [1]. Its annual incidence globally is between 0.5 to 1 case per one million individuals [2]. Its global prevalence rate is 0.03% [3]. It has a female-to-male ratio of 1.8: 1 [2]. It commonly occurs between the third and sixth decades of life, with a mean age of 50 years [1,2]. Cardiac myxomas are rarely seen at extremes of life [1,2]. Only a few cases have been reported in Africa, and its epidemiology in Africa is unknown [4,5]. Cardiac myxomas are benign, well-circumscribed solitary masses that have a predilection for the left atrium [6]. They are usually slow-growing and asymptomatic until they become large and, thus are often missed. The symptoms are non-specific and may resemble other cardiovascular and systemic diseases [7]. The clinical presentations are grouped into the triad of obstructive, embolic, and constitutional symptoms [7]. A high index of suspicion with the appropriate diagnostic tools is, therefore, required for its accurate diagnosis. The clinical evaluation and management of a rare case of left atrial myxoma with features of heart failure-like symptoms, acute pulmonary edema and syncope is presented here.

## Patient and observation

**Patient information:** a 68-year-old retired female civil servant presented with sudden onset dyspnea, cough, and loss of consciousness. The patient's difficulty with breathing started suddenly while driving, and progressively worsened over 12 hours. There was associated orthopnea, easy fatigability, and a cough productive of whitish frothy sputum. There was no history of chest pain, palpitations, leg swelling, or headache. She had a brief loss of consciousness that lasted for about 5 minutes, before her presentation. She was diagnosed with hypertension four years before her presentation. She had no previous history of diabetes.

**Clinical findings:** on clinical examination, she was found conscious, but confused and restless, with a Glasgow coma score of 12/15 (eye response=4, verbal response=2, motor response=6). Her body temperature was 37.2 Celsius. She was not pale or icteric. Her oxygen saturation was initially 64% and later 91-92% after administration of 15L/min of supplementary oxygen via a non-rebreather face mask. She had asterixis and bilateral pitting leg edema. Her respiratory rate was 36 cycles/min with widespread crepitations. Her pulse rate was 112 beats/min, regular and small in volume. Her blood pressure was 143/95 mmHg. The apex beat was heard at the fifth left intercostal space, mid-clavicular line, and S1, S2, and S3 heart sounds were heard. The abdomen was non-tender and there was no palpable organomegaly.

**Timeline of current episode:** April 21^st^, 2021: the patient was admitted to our facility. April 22^nd^-25^th^, 2021: plain chest X-ray, complete blood count, fasting lipid profile, serum electrolytes, urea and creatinine, international normalized ratio, serum viral serology, urinalysis, serum cardiac enzymes, and brain computed tomography (CT) scan were done. April 23^rd^, 2021: Echocardiogram, chest CT scan, and CT coronary angiogram were done. April 26^th^, 2021: an open-heart surgery with excision of the left atrial mass was performed. April 28^th^, 2021: a repeat plain chest X-ray was done. April 30^th^: a histology report of the surgically excised left atrial mass was released and the patient was discharged home.

**Figure 1:**
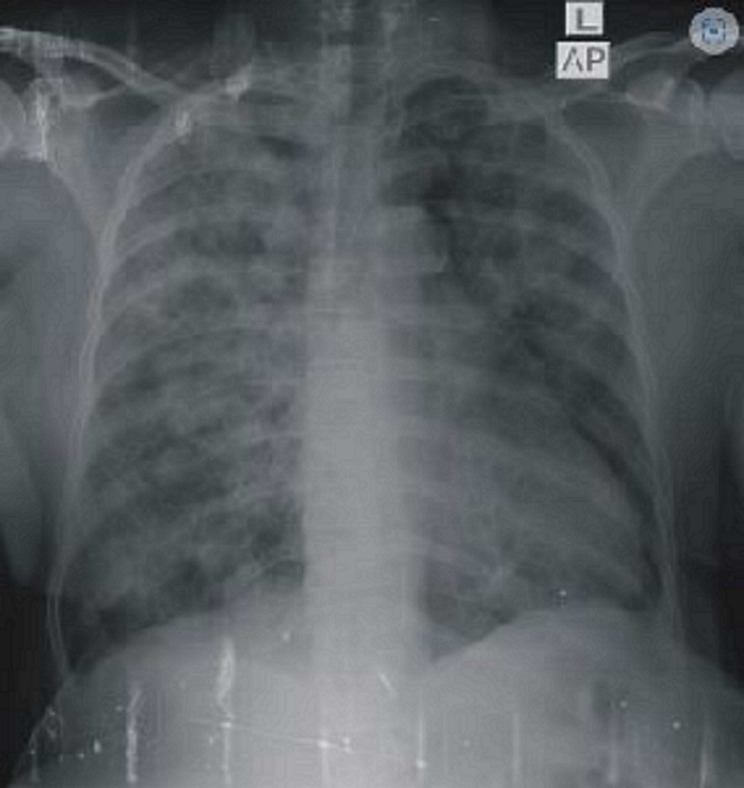
plain chest X-ray (anterior-posterior view) showing features of bilateral pulmonary edema

**Figure 2:**
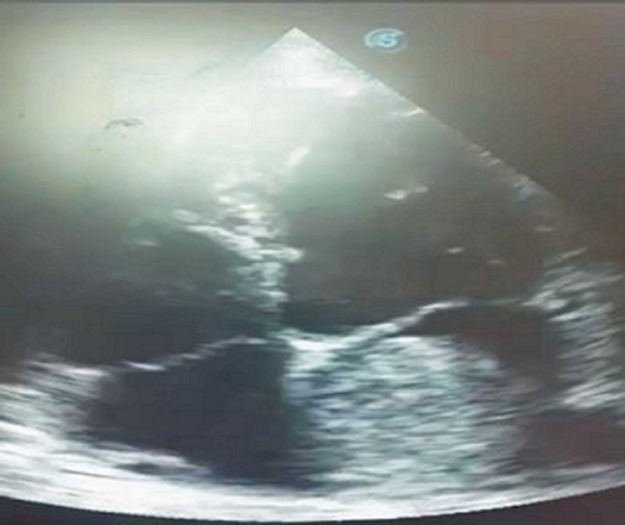
two-dimensional echocardiogram; a large left atrial mass is seen attached to the interatrial septum

**Figure 3:**
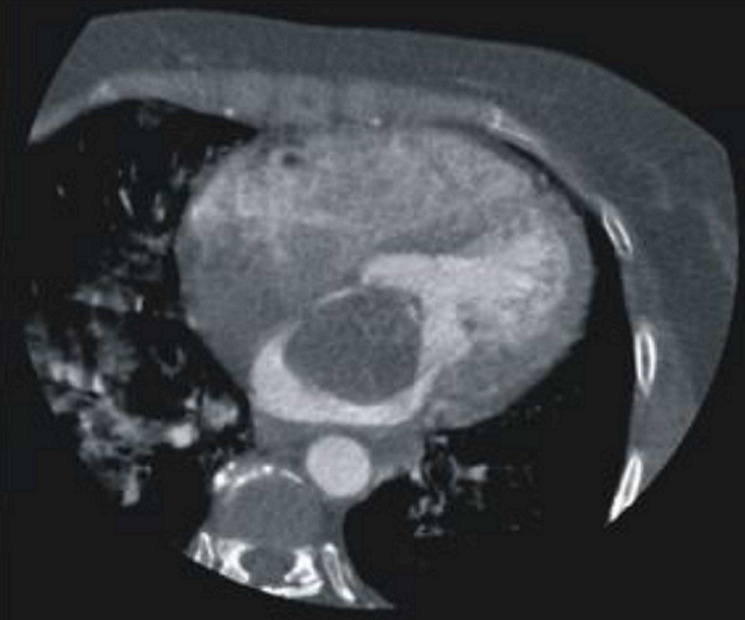
chest computed tomography (CT) scan; a left atrial mass is seen with an attachment to the interatrial septum

**Figure 4:**
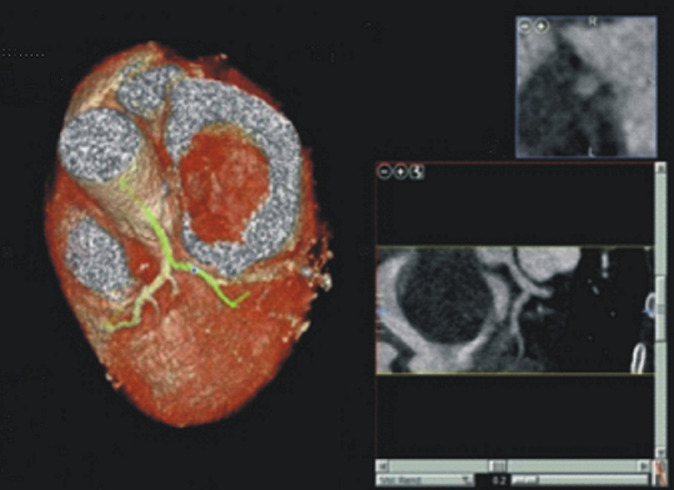
computed tomography coronary angiography, normal angiogram; a minimally enhancing hypodense lesion is seen in the lumen of the left atrium

**Diagnostic assessment:** a 12-lead electrocardiogram mainly showed sinus tachycardia. A plain chest X-ray revealed features consistent with bilateral pulmonary edema, including diffuse air-space opacities in 90% of both lung fields with perihilar haze and blunting of the left cardiophrenic angle (Figure 1). Her complete blood count showed an elevated white cell count of 22,200/mm^3^, with a relative granulocyte count of 84.9% and a lymphocyte count of 11.6%. Hematocrit was 35.8% and erythrocytes sedimentation rate (ESR) was 64 mm/hr. Her fasting serum lipid profile showed elevated lipids. Her 2-dimensional echocardiogram revealed a large left atrial mass attached to the interatrial septum, mitral regurgitation, and normal-sized ventricles with preserved ventricular function (Figure 2). Chest CT and CT coronary angiography showed a minimally enhancing hypodense mass measuring 5.8 x 3.5 x 4.3 cm (L x AP x Tr) in the lumen of the left atrium attached to its anterior wall with a padding effect on the superior part of the left ventricle and aortic root (Figure 3, Figure 4). The main coronary branches showed a normal angiogram (Figure 4). The results of other investigations were normal.

**Diagnosis:** an initial presumptive clinical assessment of acute left ventricular failure with possible congestive cardiac failure and chest infection was made at presentation. A preliminary diagnosis of left atrial mass, most probably an atrial myxoma, with features of pulmonary edema, was subsequently made following the above investigation results.

**Figure 5:**
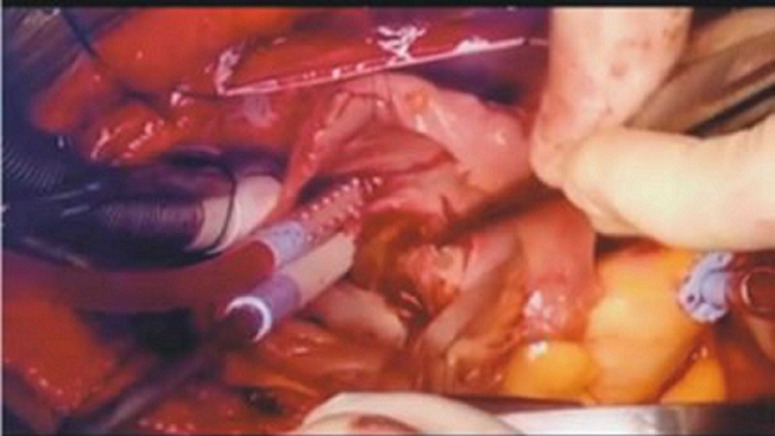
the surgical findings; a large left atrial polypoid mass is seen with its stalk attached to the interatrial septum

**Therapeutic interventions:** at presentation, she was given intravenous Furosemide 80mg stat then 40mg 12 hourly, intravenous Augmentin 1.2g stat, then 600mg 12 hourly, tabs Lisinopril 5mg once daily, tabs Bisoprolol 2.5mg once daily, tabs Rivaroxaban 15mg once daily, and tabs Rosuvastatin 10mg nocte. An open-heart surgery with excision of the left atrial mass (Figure 5) was done for the patient after informed consent was obtained from her. The surgical procedure was uneventful.

**Figure 6:**
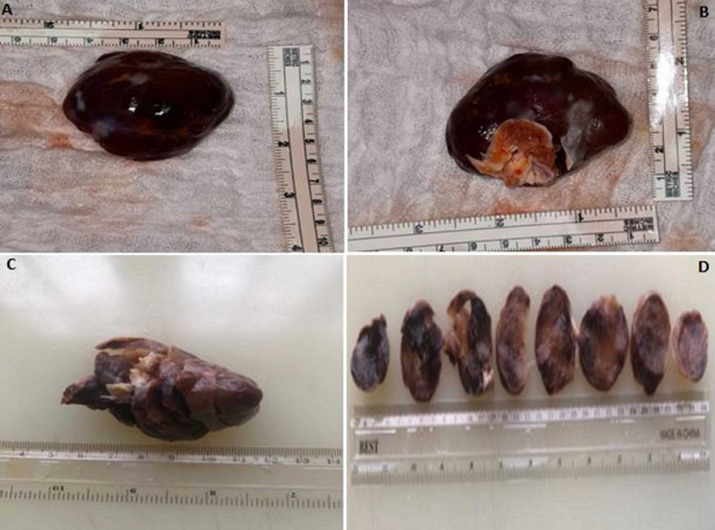
gross pictures of the left atrial mass (A, B: gross pictures; C, D: serial sections of the mass)

**Figure 7:**
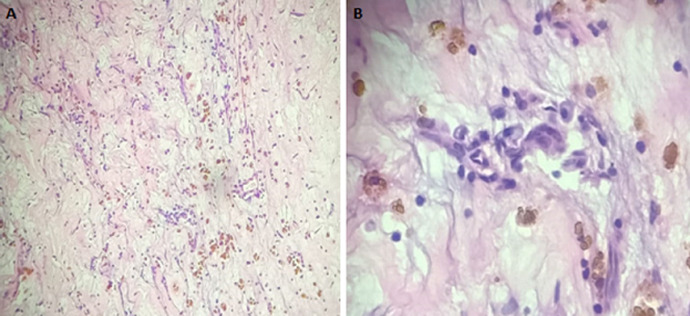
histologic photomicrographs of the left atrial myxoma (hematoxylin and eosin staining, A: x 40 magnification; B: x 100 magnification)

**Follow-up and outcome of intervention:** the patient´s condition after the surgery was stable. A repeat post-operation plain chest X-ray showed resolving pulmonary edema. Histopathologic report of the excised left atrial mass includes a gross description of the mass as an oval-shaped, brown-to-yellow, soft-to-firm, encapsulated polypoid tissue with smooth surfaces, that measures 5cm x 4cm x 3cm and weighs 5g. Histologic sections of the mass show loose clusters of benign stellate-to-globular cells that have abundant eosinophilic cytoplasm, indistinct cell borders, oval nuclei with open chromatin and indistinct nucleoli, within a fibromyxoid stroma, consistent with an atrial myxoma (Figure 6, Figure 7). She was discharged home on her antihypertensive medications and some antibiotics. Her post-operative condition was excellent. She had two-weekly follow-up visits for two months and then was followed up every two months for two years with intermittent screening for recurrence using echocardiography. She has had no recurrence.

**Patient perspective:** the medical intervention I received was timely and quite a relief. I feel much better after my discharge. I am thankful to the medical team at ABUADTH for their professionalism.

**Informed consent:** an informed consent for the publication of this article in a journal was obtained from the patient.

## Discussion

Myxomas are rare benign mesenchymal tumors that commonly affect the heart [1]. Cardiac myxoma is rare, constituting 30-50% of all primary cardiac tumors, with a global prevalence rate of only 0.03% and a female preponderance [2,3,5]. A metanalytical study showed 80% of cardiac myxomas occurred on the left wall of the left atrium near the fossa ovale, as seen in the index case [6]. The pathogenesis of cardiac myxoma is poorly understood. They are believed to arise from primitive multipotent cardiac mesenchymal cells with excessive glycosaminoglycan production [8]. Ninety percent of cardiac myxomas are sporadic. Mutations in GNAS1 gene and Protein Kinase Camp-dependent type I regulatory subunit alpha (PRKARIA) genes (Carney syndrome) have been implicated in familial cases of cardiac myxomas [8]. Cardiac myxomas, when symptomatic, may mimic various other cardiovascular and systemic diseases [7]. Our index patient presented with clinical features mimicking congestive heart failure. These clinical presentations are due to the obstruction of the left atrioventricular blood flow by the tumor. Left atrial myxoma is usually pedunculated, and when large enough, can occlude the left atrioventricular valve ("ball valve" obstruction of the mitral valve) during diastole mimicking mitral stenosis, but sways away into the left atrium during systole [6-8]. This explains the positional change in the cardiac murmur that is usually seen in left atrial myxomas. If the tumor destroys the mitral valve, the patient can present with features of mitral regurgitation [6-8]. Constitutional symptoms of fever, malaise, weakness, and weight loss are believed to be due to tumoral secretions of some chemokines including interleukin-6 that mediates an acute phase response [6-8]. Diagnosis of cardiac myxomas is usually made non-invasively using echocardiography. Radiologic findings of cardiac myxoma vary. Most of the 86 cardiac myxoma cases reviewed by Grebenc *et al*. had unusually nonspecific radiographic features [9]. They recommended the use of cine gradient recalled echo (GRE) magnetic resonance images for accurate assessment of the size, location, and point of attachment of cardiac myxomas [9]. The definitive diagnosis of left atrial myxoma in the index case was made using echocardiogram and histology. Other similar case reports of patients with left atrial myxoma presenting with acute pulmonary edema and heart failure-like features, diagnosed with echocardiography, have been reported [4,10]. Cardiac myxomas can be fatal, especially when there are complications of left atrioventricular valvular obstruction, tumor embolization, and cardiac arrhythmia [8]. With obstruction of the atrioventricular valve, it can result in acute pulmonary edema as seen in the index case. If not promptly excised, complications of the tumor may lead to sudden death. Cardiac myxomas have an excellent prognosis when detected early and completely excised. They rarely recur, except for patients with Carney syndrome [8].

## Conclusion

Cardiac myxoma is a rare tumor with variable, non-specific presentations that can mimic various cardiovascular and systemic diseases, posing a diagnostic dilemma. Here, a middle-aged African woman who had a previous diagnosis of hypertension presented with clinical features initially suggestive of an acute left ventricular failure. However, the definitive diagnosis of a left atrial myxoma was made with the aid of an echocardiogram and histology of the promptly excised left atrial mass. Cardiac myxoma is a great mimicker. However, its early detection with complete surgical excision can be curative and lifesaving. Therefore, a high index of suspicion, with the use of appropriate diagnostic tools, is essential for its accurate diagnosis and management.
